# Exercise reduces physical alterations in a rat model of fetal alcohol spectrum disorders

**DOI:** 10.1186/s40659-024-00520-2

**Published:** 2024-06-22

**Authors:** Ronald Vargas-Foitzick, Bayron García-Ordenes, Donovan Iratchet, Angie Acuña, Spencer Alcayaga, Cristian Fernández, Karla Toledo, Marianela Rodríguez, Carolina Naranjo, René Bustamante, Paola A Haeger

**Affiliations:** 1 Carrera de Kinesiología, Facultad de Medicina, Universidad Católica del Norte, Coquimbo, Chile; 2 Departamento de Ciencias Biomédicas, Facultad de Medicina, Universidad Católica del Norte, Coquimbo, Chile; 3 Departamento de Ciencias Clínicas, Universidad Católica del Norte, Coquimbo, Chile; 4 Núcleo de Investigación en Prevención y Tratamiento de Enfermedades Crónicas no Transmisibles (NiPTEC), Universidad Católica del Norte, Coquimbo, Chile

**Keywords:** Fetal alcohol syndrome, Prenatal alcohol exposure, Endurance training, Enriched environment, Resistance training, Adolescents, Strength, Physical capabilities

## Abstract

**Background:**

Prenatal alcohol exposure (PAE) has serious physical consequences for children such as behavioral disabilities, growth disorders, neuromuscular problems, impaired motor coordination, and decreased muscle tone. However, it is not known whether loss of muscle strength occurs, and which interventions will effectively mitigate physical PAE impairments. We aimed to investigate whether physical alteration persists during adolescence and whether exercise is an effective intervention.

**Results:**

Using paradigms to evaluate different physical qualities, we described that early adolescent PAE animals have significant alterations in agility and strength, without alterations in balance and coordination compared to CTRL animals. We evaluated the effectiveness of 3 different exercise protocols for 4 weeks: Enrichment environment (EE), Endurance exercise (EEX), and Resistance exercise (REX). The enriched environment significantly improved the strength in the PAE group but not in the CTRL group whose strength parameters were maintained even during exercise. Resistance exercise showed the greatest benefits in gaining strength, and endurance exercise did not.

**Conclusion:**

PAE induced a significant decrease in strength compared to CTRL in PND21. Resistance exercise is the most effective to reverse the effects of PAE on muscular strength. Our data suggests that individualized, scheduled, and supervised training of resistance is more beneficial than endurance or enriched environment exercise for adolescents FASD.

**Supplementary Information:**

The online version contains supplementary material available at 10.1186/s40659-024-00520-2.

## Background

Alcohol is a teratogen, a toxic substance that induces congenital defects during pregnancy [1]. Prenatal Alcohol Exposure (PAE) has important consequences for cognitive, neurological [1], and muscular [2] development of progeny. The dysfunctions associated with PAE are known as fetal alcohol spectrum disorder (FASD) which is characterized by a variety of developmental, cognitive, and behavioral abnormalities [3]. Alcohol alters the placentation of rats in a dose-dependent manner, leading to altered fetal growth and birth weight [2]. Deprivation of the fetus of nutrients and growth factors during myofiber formation can have lasting impacts on the number of myofibers and postnatal muscle growth [2, 4].

PAE alters the coordination, motor response, and balance [5, 6] most likely to smaller fibers on the FASD model persistent until PND35 consistent with low body weight [7]. FASD manifests hypotonia and flaccidity in infants [8]. FASD child manifest deficits in the regulation of isometric and isotonic force [9, 10]. This can lead to disturbances in gross motor skills, which use large muscle groups for coordinated body movements such as walking, running, jumping, and the maintenance of balance (Review in Lucas et al. [6]).

Clinical analysis of FASD children under 13 years old has shown alterations in coordination and balance, directly associated with the mother’s alcohol consumption levels [11]. However, other authors only have been reported the association of FASD with lower scores in visual-motor integration but not with balance, coordination, or other motor skills [12]. The full understanding of the mechanism of impairment in motor skills in adolescent FASD is needed. Still, there is consensus that gross motor assessment and intervention strategies are necessary to improve disability in FASD children [11, 12].

Exercise seems to be effective for FASD and other disorders. In this sense, it has been described that exposition to voluntary resistance exercise attenuated PAE-induced spatial learning and memory deficit in PAE rats in adolescence period (PND54) [13]. Moreover, voluntary exercise from PND21 to PND51 of rats exposed to alcohol during the first postnatal days, ameliorated in memory deficit [14]. In humans, exercise mitigated motor impairments in adolescents with spinal muscular atrophy, resulting in increased strength and motor function after 12 weeks of progressive resistance training [15]. Moreover, 10-week of motor activity program (body awareness, motor planning, bilateral motor balancing skills, fine motor coordination and visual-moto performance) improved the balance, strength, and coordination in adolescents with autism [16]. Endurance exercise also improves behavioural alteration (spatial memory and anxiety) caused by PAE [13, 14, 17].

Muscle contraction generated by exercise, can induce release the different myokines [18]. For example, irisin is a hormone that is secreted mainly at the skeletal muscle level as a response to different modalities of exercise such as high intensity interval training, resistance and endurance exercise [19–21]. Since irisin has neuroprotective function improving cognitive functions of memory and learning [21, 22] demonstrating causality by blocking in vivo irisin action using specific antibodies [21], we hypothesized that could have a role in FASD because also has cognitive deficits as we described above.

PAE generates alterations at the muscle level and in executive function [9–13]; however it is unknown whether different protocols of voluntary or programmed exercises may be beneficial in the muscular response to tests of strength, coordination, agility, or balance in adolescents PAE.

The purpose of this study is to investigate whether physical disabilities persist during adolescence and to evaluate different protocols of exercise as a therapeutic intervention in a rat model of FASD.

## Results

We developed an animal FASD model which feature is shown in Table 1. The mothers exposed to ethanol drank pharmacologically relevant concentrations of ethanol (3.96 ± 0.43 g/kg/day), compared to the CTRL mothers. Additionally, they drank significantly less total liquid (total liquid consumed (mL), with or without ethanol) over the experimental period (t10 = 6.00, p = 0.000026); however, the additional water compensates for this difference. Additionally, the ethanol-exposed mothers had no significantly lower body weight during pregnancy (p = 0.52). The weight of the offspring was evaluated at birth. On average control newborn litters weighed 6.85 ± 0.11 g (N = 4 litters), and PAE animals had an average weight of 6.58 ± 0.21 g (N = 9 litters). Litter size and offspring weight in post-natal day 1 (PND1) did not differ between conditions. Since there were no significant differences in birth weights, these results are strongly suggesting that these individuals do not present FAS but are within the FASD spectrum mainly for the normal weight of newborn (Table 1).

**Table 1:** Effects of forced ethanol (10%) consumption on rat dams and their offspring

Variable	Maternal treatment		P value
	Ethanol	Water	
Maternal ethanol consumption (g ethanol/kg/day)	3.96 ± 0.43	NA	NA
Maternal liquid consumption (ml/day)	23.61 ± 1.55	39.88 ± 1.30	< 0.001
Maternal additional water consumption (ml/day)	19.96 ± 0.02	NA	NA
Average of maternal weight (g)	338.14 ± 4.82	342.50 ± 3.74	0.528
Offspring weight-postnatal 1 day (g)	6.58 ± 0.21	6.85 ± 0.11	0.325

Both treatment include sucralose 64 mg/l. P, statistical probability

Analysis by Student’s t-test (p value). Mean ± SEM, CTRL (N = 4); PAE (N = 9). NA, not applicable

In this study agility, and strength were evaluated at the time of weaning (PND21) and in PND50 after 4 weeks of the exercise protocols. To evaluate strength in animals we measured the hanging time from the inverted mesh, a test validated by Deacon which is effective with minimal influence of others factors such as coordination [23], in addition it has been used effectively for others authors to measure strength [24, 25]

### Effects of PAE on the physical qualities of the pups on early adolescent period (PND21)

**Fig. 1:**
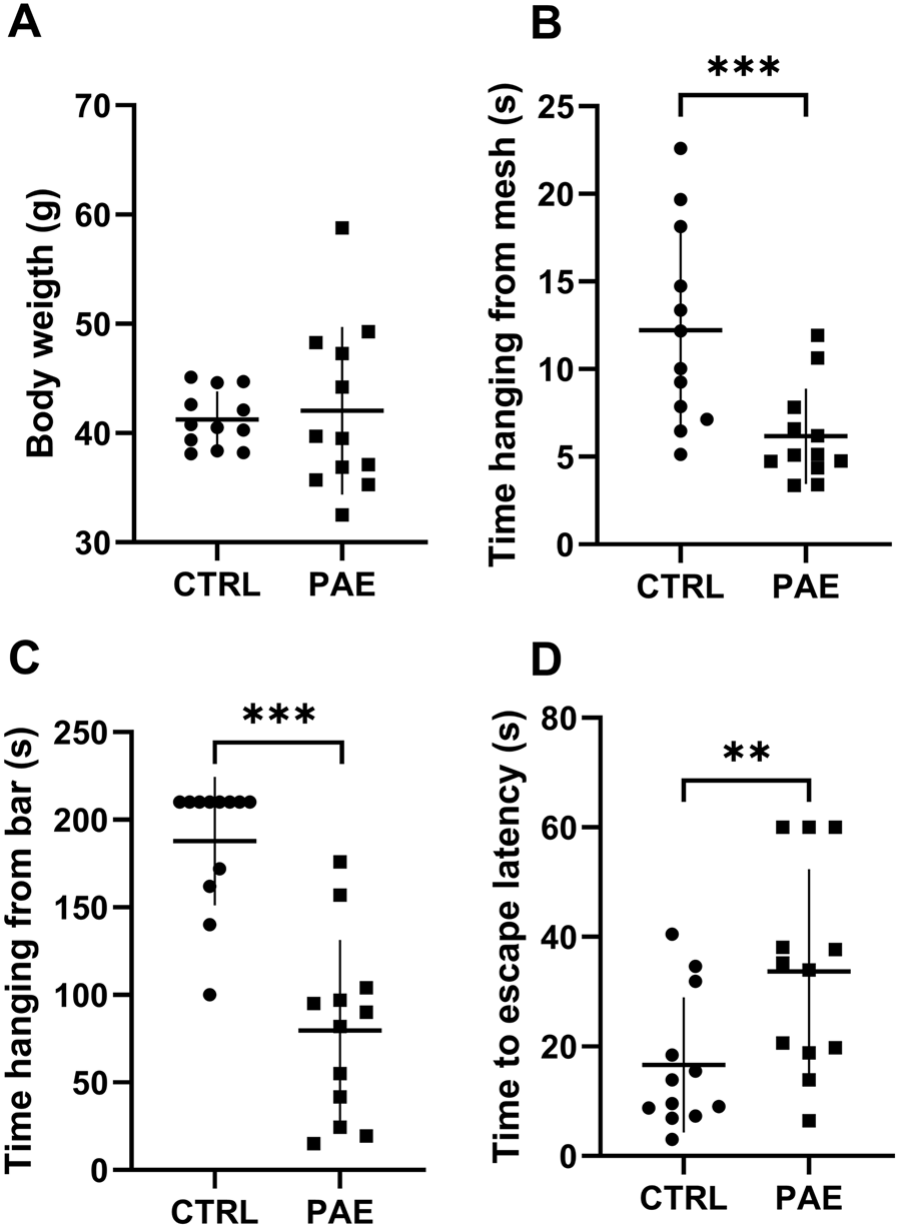
PAE affects physical capabilities at PND21. Prenatal alcohol exposure leads to a decreases hanging time from mesh and bar before the exercise protocol. **A** Body weight at PND21 (CTRL N = 12; PAE N = 12 animals). **B** Hanging Time from Mesh (CTRL N = 12; PAE N = 12 animals). **C** Hanging time fro bar (CTRL N = 12; PAE N = 12). **D** Time to latency to Scape (CTRL N = 12; PAE N = 12). Data are mean ± SEM. * p < 0,05 ** p < 0,01 *** p < 0.001 (Mann–Whitney Test)

As shown in Fig. 1, exposure to alcohol during pregnancy impacts the physical qualities of the pups. Body weight evaluation in PND21 revelead no significant difference between CTRL and PAE groups (41.23 ± 0.74 g and 42.05 ± 2.21 g; p = 0.7125) (Fig. 1A). Hanging time from the inverted mesh was significantly lower in PAE compared to control rats (12.21 ± 1.62 s and 6.17 ± 0.78 s; p = 0.0012, CTRL and PAE respectively, Mann–Whitney test) (Fig. 1B). We were also observed same significant differences in the time hanging from the horizontal bar test (187.8 ± 10.61 s and 79.73 ± 14.93 s; p < 0.0001, CTRL and PAE, Mann–Whitney test) (Fig. 1C). Moreover, PAE animals exhibited a longer time to complete the movement on the elevated bar compared with control rats, suggesting alterations in agility performance(16.62 ± 3.56 s and 33.70 ± 5.39 s; p = 0.015, CTRL and PAE, Mann–Whitney test) (Fig. 1D). This data suggest that PAE offspring have alteration on physical qualities on early adolescent period..

Others physical qualities of PAE and control animals were measured. The balance test showed no significant differences between the groups on the bar performance, concerning with their number of stops (1.37 ± 0.47 and 1.91 ± 0.51; p = 0.45, CTRL and PAE, Mann Whitney test) (Supplementary Fig. 1A); slips (0.75 ± 0.31 and 1.20 ± 0.28; p = 0.1082, CTRL and PAE, Mann Whitney test) (Supplementary Fig. 1B) and time (30.27 ± 5.5 and 16.60 ± 3.6; p = 0.0761, CTRL and PAE, Mann Whitney test) (Supplementary Fig. 1C). When analyzing the data obtained in the coordination test, significant differences were found in the parameters of front stride length (5.00 ± 0.05 cm and 4.78 ± 0.01 cm; p = 0.0024, CTRL and PAE, Mann–Whitney test) (Supplementary Fig. 1D) but not on hind stride length (4.97 ± 0.072 cm and 4.82 ± 0.07 cm; p = 0.1089, CTRL and PAE, Mann–Whitney test) (Supplementary Fig. 1E). Similarly, significant differences in front step width were found (1.70 ± 0.03 cm and 1.64 ± 0.06 cm; p = 0.32, CTRL and PAE, Mann–Whitney test) (Supplementary Fig. 1F) but not found on hind step width (3.38 ± 0.04 cm and 3.28 ± 0.05 cm; p = 0.1558, CTRL and PAE, Mann–Whitney test) (Supplementary Fig. 1G).

### Effect of 4-week enriched environment on adolescent rats (without and with PAE)

**Fig. 2:**
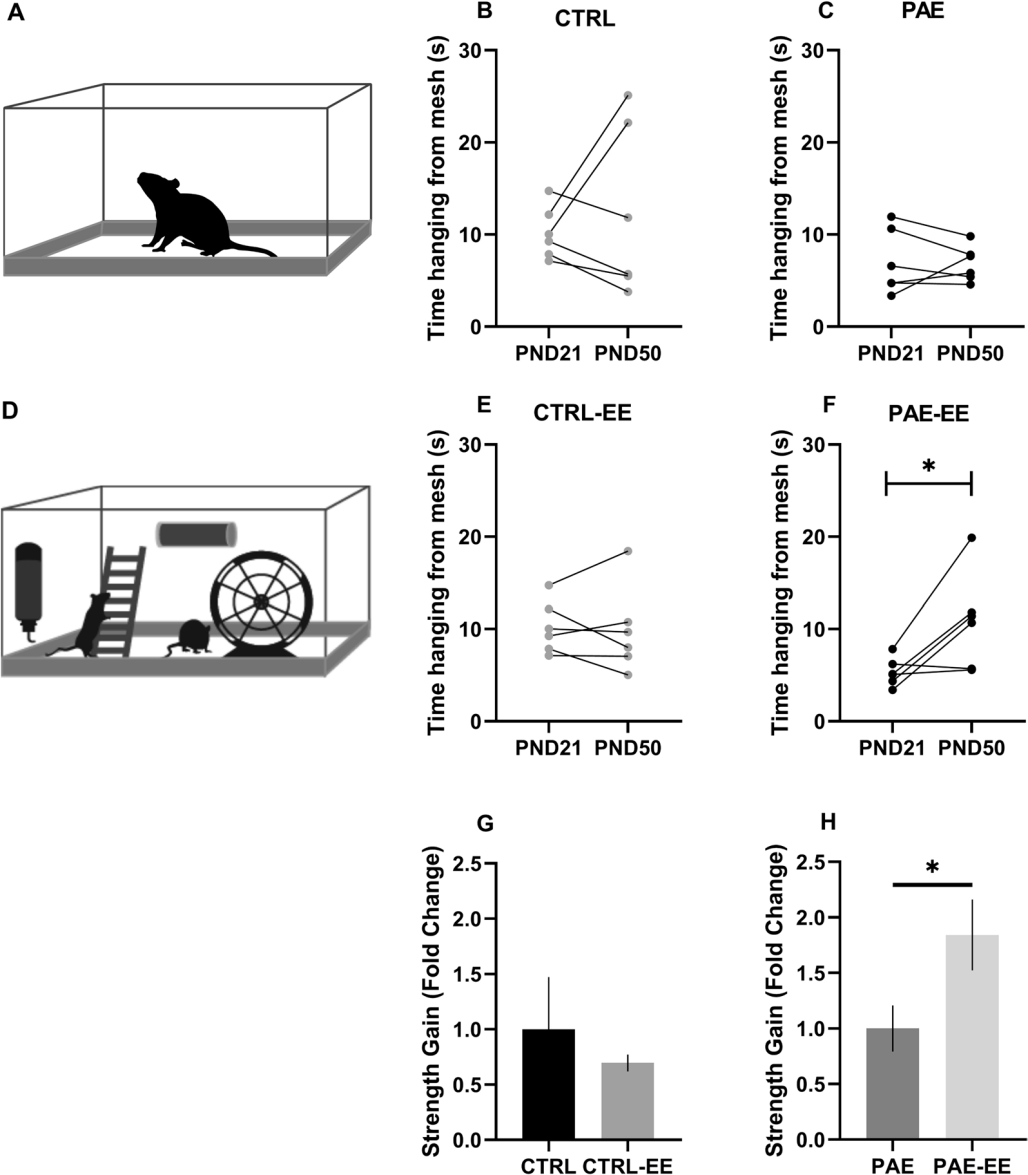
Effects of an enriched environment on hanging time from mesh. Hanging time increased only in the PAE group exposure to an enriched environment for 4 weeks. **A** Control condition scheme. **B** CTRL group without exposure to an enriched environment in hanging time from mesh. **C** PAE group without exposure to an enriched environment in hanging time from mesh. **D** Enriched environment scheme **E** CTRL group with exposure to an enriched environment in hanging time from mesh. **F** PAE group with exposure to an enriched environment in hanging time in mesh (Wilcoxon test). G and H. Fold of changes in the strength gain (time hanging on the mesh of animal after respect before intervention) respect to CTRL (**G**) or PAE (**H**) Mann–Whitney Test. N = 6 animals per group. Data are mean ± SEM * p < 0.05

First at all we measured the effect of enrichment environment training. To determine the effectiveness of EE, we re-evaluated strength after 4 weeks in adolescent animals (PND50) (Fig. 2). As control of the protocol, we used a sedentary group (CTRL and PAE) that lived in conventional cages (Fig. 2A). Other groups were lived in EE (CTRL-EE and PAE-EE) (Fig. 2D). When comparing the groups before and after the periods of training protocol on hanging time from the inverted mesh, we did not find significant differences between both ages (10.20 ± 1.15 s and 12.35 ± 3.75 s, p = 0.5, CTRL group, Wilcoxon test) (Fig. 2B) and (7.01 ± 1.46 s and 6.85 ± 0.78 s, p = 0.3438, PAE groups, Wilcoxon test) (Fig. 2C). Similarly, CTRL-EE did not have significant differences in PND21 and PND50 (10.20 ± 1.57 s and 9.83 ± 1.97 s, p = 0.34, Wilcoxon test) (Fig. 2E). However, group PAE-EE, did show significantly higher time hanging from the inverted mesh after the protocol (5.33 ± 0.62 s and 10.85 ± 2.13 s, p = 0.046, Wilcoxon test) (Fig. 2F). These analyses suggest that growth and weight do not influence the hanging time gains demonstrated in sedentary groups. Additionally, to determine the change in strength, we calculated the strength gain by measuring the time hanging from the inverted mesh of the animal after respect to before the intervention, following calculation of fold of changes respect to groups without exercises. CTRL and CTRL-EE did not show statistical significant differences (1 ± 0.4727 and 0.6960 ± 0.07581, p = 0.6991, Mann–Whitney test). PAE-EE showed a statistical significant increase in strength gain compared to PAE [1 ± 0.2081 (PAE) and 1.843 ± 0.3196 (PAE-EE), p = 0.0411, Mann–Whitney test]. The results suggest that the EE could reverse the results found in PND21 in PAE animals.

Since in the enrichment environment the exercise of animals is voluntary, we could not control the time spending in the exercise or the specific was not possible to evaluate the kind of exercise performed, we decided to evaluate each separated in a controlled manner. We trained animals in endurance training forced to run a wheel for 20 min a day, or in resistance exercise forced to climb a ladder with weight in the tail.

### Effects of endurance exercise on the strength of adolescent rats

Group PAE with endurance exercise (PAEEX), did not show significant differences in the time hanging from the inverted mesh after a 4-week of training (4.10 ± 0.54 s and 14.15 ± 5.23 s, p = 0.062, PND21 and PND50, Wilcoxon test) (Supplementary Fig. 2A). Subsequently, we continue the training for two more weeks, and time hanging was re-evaluated. We didn’t observe a difference in time of hanging at 6 weeks compared to the pre-training period (4.10 ± 0.54 s and 7.92 ± 0.67 s; p = 0.0625 PND21 and PND64, Wilcoxon test) (Supplementary Fig. 2B) suggesting no change in strength with this protocol assessed with the inverted mesh test. To assess if there was a correlation between the amount of exercise performed and the acquisition of strength, we compared the average number of laps performed by the animal in PND50 and PND64 and compared to its strength. No significant correlation was found between the number of revolutions per minute (RPM) and the time achieved in the test (r = 0.42, p = 0.28, Pearson correlation test) (Supplementary Fig. 2C). These results suggest that endurance training for 4 or 6 weeks on the wheel running was not as effective in the strength gain evaluated over time on hanging from the inverted mesh test.

### Effects of resistance exercise in the strength on adolescent rats

**Fig. 3:**
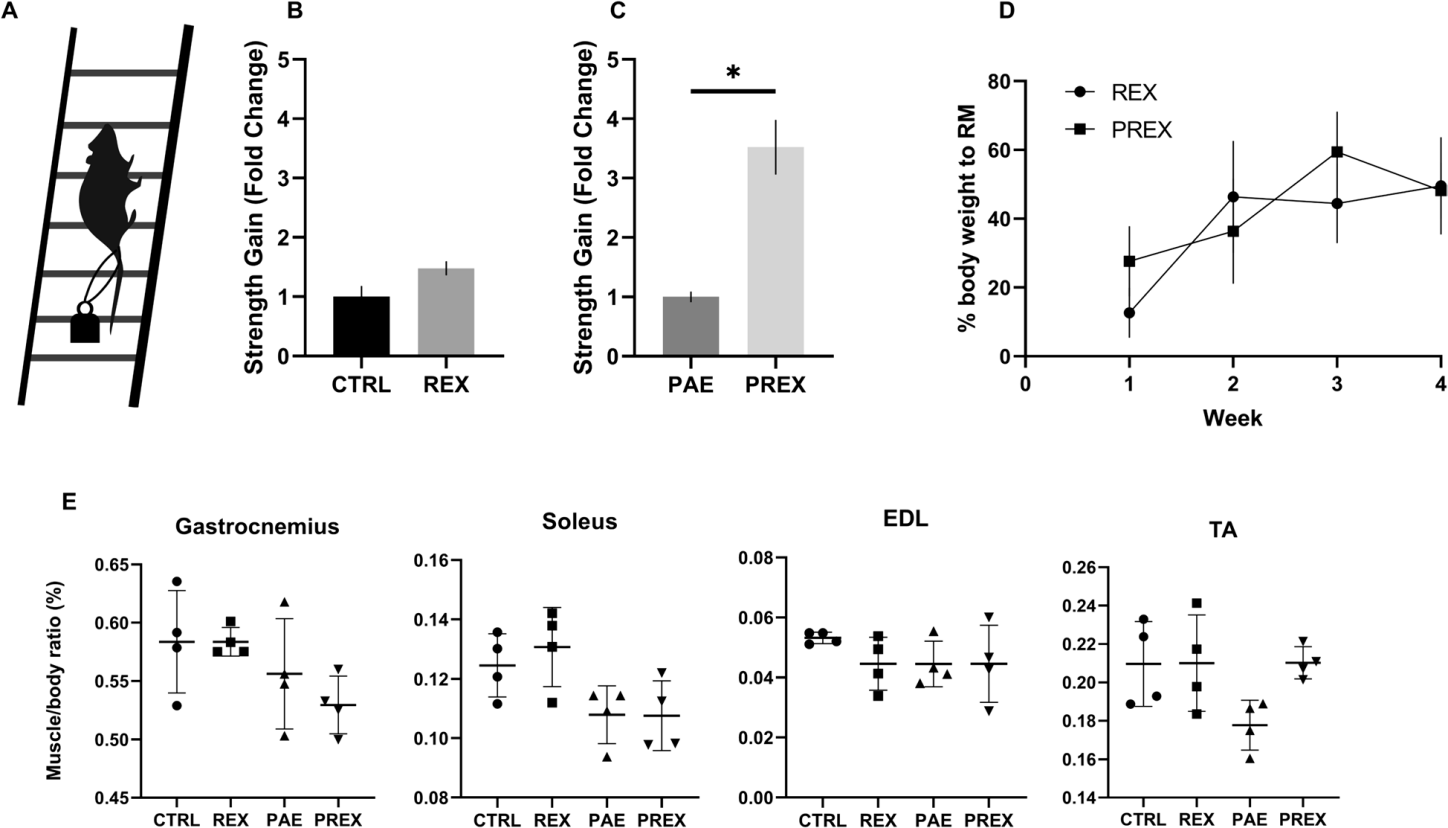
Effects of resistance exercise on hanging time from mesh in adolescent rats. Resistance exercise for 4 weeks increased hanging time evaluated in bar and mesh test only in PAE animals. **A** Resistance exercise scheme. **B**, **C** Fold of changes in the strength gain (time hanging on the mesh of animal after respect before intervention) respect to CTRL (**B**) or PAE (**C**) (Mann–Whitney test). **D** Strength progression during exercise protocol. **E** Muscle weight expressed as a percentage of the total body mass (Gastrocnemius, Soleus, Extensor Digitorum Longus and Tibialis anterior) (Kruskall-Wallis test). N = 4 animals per group. Data are mean ± SEM. * p < 0.05 (Mann–Whitney test)

The time of hanging from the inverted mesh was measured after 4 weeks of resistance exercise and compared with the CTRL group (Fig. 3). To determine the effect of strength training under controlled conditions, rats were trained for 1 month with the controlled REX by climbing the ladder with a weight hanging from the tail (Fig. 3A). In PND50 we observed a robust statistical significant difference on strength gain (around fourfold of change) in PREX respect to PAE group (1 ± 0.09152 (PAE) and 3.522 ± 0.4593 (PREX), p = 0.0286, Mann–Whitney test)(Fig. 3C). CTR and REX, didn´t shown a difference on strength gain (1 ± 0.4199 and 1.479 ± 0.1189, p = 0.0571, Mann–Whitney test)(Fig. 3B), as evaluated on hanging from the inverted mesh test. To evaluate the progression of exercise resistance, the maximal RM was measured with respect to body weight in each session in REX and PREX animals, a significant difference was found between weeks (p = 0.045) but not difference in groups (p = 0.7825) (Fig. 3D), suggesting that the training was effective for both groups. To analyze the exercise effects on the gain in muscle size, we weighed the gastrocnemius, soleus, EDL, and tibialis anterior in rats with and without exercise. We didn´t observe a difference between groups in Kruskal–Wallis analysis (Fig. 3E, Supplementary Table 1). These results suggest that resistance exercise for only 4 weeks with individualized and progressive loading can be effective for increasing the strength gain associated to hanging time in PAE without change in muscle mass in both groups.

### Effects of resistance exercise in the irisin levels on adolescent rats

**Fig. 4:**
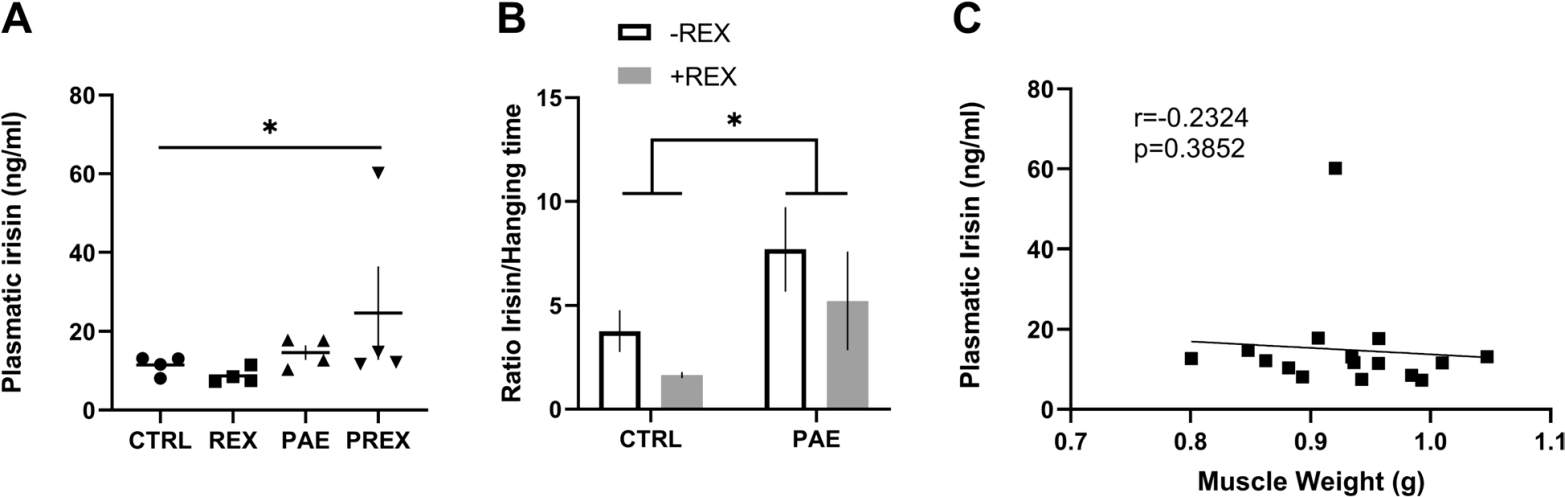
Effects of resistance exercise in the irisin levels on adolescent rats. Resistance exercise increased irisin levels in PAE after 4 weeks. **A** Irisin levels in plasma (ng/ml) (Kruskall Wallis Test). **B** Irisin levels in plasma to hanging time by the mesh; white bar represents without exercise and grey bar represents with exercise (Two-way ANOVA). **C** Spearman’s correlation analysis between Plasmatic Irisin concentration and total right limb (leg) muscle weight post-exercise (N = 4 animals per group). Data are mean ± SEM * p < 0.05

To understand the mechanism behind resistance exercise to improve strength in PAE rats, we measure the levels of the myokine Irisin in plasma after resistance exercise for 4 weeks. When comparing group control and PAE after exercise and did not find significant differences (11.5 ± 1.8 ng/ml, 8.69 ± 0.96 ng/ml, 14.65 ± 1.85 ng/ml, 24.71 ± 11.85 ng/ml, CTRL, REX, PAE, PREX, respectively, p = 0.041, Kruskal–Wallis test), and post hoc test, did not find differences to compare different groups (Fig. 4A). When we normalized the irisin levels with the hanging time from the inverted mesh, PAE shown higher irisin levels than CTRL (F (1, 12) = 5.233, p = 0.041) but not between the 4 groups (Fig. 4B). Spearman´s correlation comparing irisin level with strength gain shown not significatively correlation (p = 0.3852; r = − 0.2324) (Fig. 4C). This analysis is suggesting that PAE increases the levels of Irisin, but not the exercise.

## Discussion

We showed that PAE decreases strength and agility in adolescent Sprague Dawley rats, however, we did not observe differences in coordination and balance in PND21. Moreover, this is the first study showing the effectiveness of exercise can mitigate motor impairment in PAE adolescent animals. We analysed three different exercise protocols and concluded that enriched environment, a free and uncontrolled physical activity, resulted in strength gain. However, we determined that the most effective intervention was a specific training protocol of resistance exercise, probably due to the progressive and individualized increase in load, as shown in training studies, ladder-based resistance training for rodents [26–28].

The PAE-induces impairment on strength and agility observed in these results could be explained by for a delay in the maturation process in the nerve-muscle junction, features that have been shown at PND14 and PND21 in PAE rats [7]. In fact, it has described that PAE subjects have predominant type II fibers over type I [2], that explain the hypotonia [29]. Moreover, this is consistent with the early fatigue in the maintenance of basal tone which affects the posture of subjects with FASD [2, 9], as well as the decreases in isotonic and isometric strength observed in subjects with FASD [9, 10]. Furthermore, ethanol is a teratogen for the development of myocytes, which can alter the organization of sarcomeres and myofilaments, resulting in embryopathic cytoskeletal dysgenesis, caused by dysplasia in the muscle structure rather than by degeneration, since there is no inflammatory response or fibrotic [2, 7, 29].

We did find significant differences between the PAE and CTRL groups only in the strid length and width (front legs) on the footprint test in the pre-adolescent stage, but not in other parameters of evaluation; this could suggest that there is an alteration in coordination or balance during gait but our results did not clearly indicate so. Contrary to our results, neonatal exposure to alcohol for 8 days after PND4, (in the accelerated maturation stage of the brain), a decrease in stride length was found in the PND21 stage in the group exposed to 2.5% w/v EtOH [30]. Similarly, another study showed that neonatal alcohol exposure alters coordination, associated with a decrease in the size of the cerebellum, more evident in males in PND21 [31]. Even though it has been described that young adult rats, exposed to the ethanol during pregnancy [32] showed cerebellar ataxia and gait dysfunction. Also, it has been described the cerebellar ataxia is related to a shortening in the length of the stride and the angle of step increases [33], however we cannot discard the cerebellum impairment by alcohol in our model. Moreover, we cannot discard that PAE also affect others neural structures affecting motor skills. For example, PAE can cause damage in structures such as the corpus callosum [34], the motor cortex [35], as well as in the peripheral nervous system [7], which are closely related to alterations in cognition, coordination, motor response, and balance. The potential abnormalities in brain regions due to PAE may represent a mechanism by which motor skill learning deficits arise, as demonstrated by accelerated rotarod and single pellet reaching tests [36]. This discrepancy in our results compared to other studies is due to the fact that some studies implemented a daily ethanol intake protocol starting at PND4 [30, 31] and in another study, higher doses of ethanol were used in rats (35% w/v of prenatal ethanol) [32].

The present study also demonstrated that physical exercise in PAE animals is an effective method for improving strength without change in muscle mass. In this regard, PREX significantly improved results in the horizontal bar and inverted mesh tests and increased carrying load because of stair climbing training. Duncan et al. [26], also showed that strength increases significantly after stair climbing training at 4 weeks in the absolute mass lifted. After 26 weeks of training they showed an increase in the cross-sectional area of the muscles of the hind limb [26].

Interestingly we did not find change in muscle strength in the control animals, which is consistent with a previously reported study using a similar protocol (8 weeks stair climbing training period)-the authors did not observe changes in muscle mass and strength on Sprague–Dawley rats [37], suggesting that this protocol will be effective only in a condition of physical alteration, as seen in PAE animal.

The observed change in PAE, in contrast to control animals, aligns with the concept of hormesis which refers to a biological phenomenon wherein exposure to low doses of potentially harmful stressors yields beneficial effects. Regular exercise serves as an intermittent stressor that promotes adaptation, but if the stress does not reach a threshold, adaptation will not occur [38, 39]. In the case of low-level physical conditions, as observed in PAE, low-level exercise loading can be effective in improving physical qualities, but in CTRL animals, this loading could prove ineffective.

It is important to study the musculoskeletal adaptations and the secretion of different substances when exercising, since one possibility is that every time exercise is performed, an adaptation occurs, requiring fewer concentrations of said substance to have relevant effects on any tissue and serious. It is relevant to study the concept of hormesis and its implication in myokines [38]

Irisin is a myokine secreted by the endocrine property of skeletal muscle to different modalities of exercise or muscle contraction [40]. In healthy mice model with endurance exercise for 8 weeks showed an increased irisin level in the exercise group [21]. Elevation of irisin levels after exercise also has been described, in humans [41] and rodent [42], but it has been observed that in physically active men irisin level decreases compared with sedentary [21]. However, has not been measured in PAE condition. Jiang et al. [43], recently described that the irisin level in serum, as well as brain irisin levels decrease proportionally with time of ethanol consumption and even with the percentage of ethanol preference, moreover the levels of serum irisin have statistically significant correlation with poor performance in cognitive test (depressive and anxiety-like behaviors). Since PAE animals, especially the model that is used in this article, have cognitive alteration [44–46], together with alteration in motor skills, we hypothesize that PAE decreased irisin levels in a basal state. Contrary to our hypothesis, we observed weak but significantly higher levels of plasma irisin in PAE compared with control animals, however, resistance exercise, which improve the strength, does not change the levels in control or PAE animals. Futures studies will be conduced to analice others posible temporal window where Irisin rose because it has been described as a transient increase of the levels of Irisin after endurance exercise and is depending of the intensity of the exercise. Huh et al. [47] described that irisin levels increased immediately after high-intensity interval exercise and declined to basal levels 1 h thereafter. Furthermore we can not discart the involvmente of another myokines in the alteration of motor skills in PAE animals as well as the exercise-dependent improvement in motor as well as cognitive skills [18, 48, 49].

Even thought, our results shown that endurance training for 4 weeks was not effective in gaining strength in adolescent rats, if we found changes, with increasing training volume (2 more week of training). This could be due to the lack of individualization of the load and its progression, which may be necessary to obtain changes in the individual performing the exercise. In this regard, a study showed that rats exposed to a wheel for 24 h for 12 weeks, showed changes at the metabolic level of muscles such as the tibialis anterior and soleus [50]. The latter also showed a significant increase in the amount of type 1 fibers and its capillarization [50]; which could be expressed as an increase in muscular endurance. This study showed no major differences between 6- and 12-week wheel training periods. However, there was an increased amount of type 1 and type 2 fibers in the control group, and increased sensitivity of fast-twitch fibers to calcium, which could produce 20% to 30% more force against the same concentrations of cytosolic calcium. It has previously been observed that voluntary wheel exercise for 6 and 12 weeks is enough to generate biochemical modifications, leading to improved muscle plasticity [50]. Therefore, it could be that we did not observe significant improvements in strength since the daily training session was short (20 min) in comparison with other protocols where they were permanently exposed to a wheel and performed voluntary wheel exercise during long periods. Likewise, another factor that could be important is the time of day in which the subjects trained, so their voluntary locomotor activity could have been decreased. However, in our study, in 4 weeks we did observe non-significant improvements induced by exposure in PAEEX and increased strength after two additional weeks of training on the running wheel. Therefore, we believe that in our case the training load density might not have been adequate to obtain significant changes.

Our study provides a basis for resistance and endurance training in PAE subjects, which generates an increase in physical qualities. In the enriched environment protocol, increases in time in hanging were observed, however, we observed greater beneficial effects after resistance training (climbing stairs) as the load was graded and individualized each week. Likewise, exercise with wheels showed positive results. These were not significant after 4 weeks but were after 6 weeks of training. We propose that in future studies, a wheel training protocol should be carried out considering the training schedule and the time of exposure of the rat to the wheel to quantify the load.

The possible limitations of the present study are the number of individuals per group, even thourth the size effect was calculated, since perhaps the number limits the observation of more robust significance of the study. In adition, for the future experients will be consider to test PAE as well exercise effect on the body composition (e.g.muscle and fat composition) and possible muscle histological alterations. Have a more accurate variable to quantify the intensity of the exercise in an animal model, to know if the necessary thresholds were reached to consider that this group performed strength exercise. Since it has been seen that 12 continuous weeks of exercise are required to have clinically significant effects [51], we predict even more robust resulted will be obtained considering longer intervention of exercise.

## Conclusion

In conclusion, the most effective protocol for strength gain was resistance training due to the characteristics of the work and its individualization. It is important to mention that in this study motor skills had no difference between CTRL and PAE animals, however, it is an aspect to be considered in PAE subjects since it generates alterations in the physical capacities of these individuals. It is essential to generate a program of intervention that comprises specific and graduated physical exercise in children or adolescents exposed to ethanol during pregnancy as a therapeutic method to mitigate the deleterious effects of teratogens during the fetal period. Finally, It is very well known that PAE produce cognitive impairments (e.g. reference [13, 14, 52], working [53] and spatial memory [14, 52]. The benefits of exercise in the central nervous system (CNS) during different stages of life have been widely demonstrated [14, 16, 17, 54–56]; as well as dramatically improve both behavioural learning and LTP induction capacity in animals after PAE [13]. Future studies will focus to understand whether exercise could be used as an integral therapy for FASD improving cognitive and physical performance.

## Methods

### Animals and treatments

13 Pregnant Sprague–Dawley albino rats were used for this study. The animals were kept under a 12 h/12 h light/dark cycle (lights on at 7:00 a.m.). All procedures were approved by the Institutional Bioethical Committee of the Department of Biomedical Sciences and complied with the Declaration of Helsinki and the Guide for the Care and Use of Laboratory Animals as promulgated by the National Institutes of Health and the European Union. These pregnant rats were treated with ethanol and others without ethanol. Food and water were administered ad libitum.

From the offspring, we used 52 male Sprague–Dawley rats, which were divided into 7 groups: Control and PAE without exercise (CTRL and PAE), enriched environment (EE)(CTRL-EE and PAEE), endurance exercise (PAEX) and resistance exercise (REX and PREX) from the nine PAE litters and four CTRL litters. On PND21, force assessment was performed on all groups using the barr and inverted mesh test.

The sample size calculation of each intervention was analyzed according to Arifin [57]

### Alcohol exposure procedures

We followed the procedures described by Contreras et al. [46] and Fernández et al. [45]. Female Sprague–Dawley rats were exposed to water or alcohol (10% v/v), both sweetened with 64 mg/l sucrose, during pregnancy and during the first 7 days of lactation (P7). The females exposed to alcohol received the alcohol solution as their only fluid for 22 h. For the remaining 2 h of each day, and to avoid alcohol-induced dehydration, they received a bottle filled with 20 ml of tap water. The amount of food (g), liquid consumption (ml), and the weight (g) of each female was recorded daily. Ad libitum access to water and food was provided from P8 to the end of the procedures. The weight of the puppies was recorded at PND 1, 7, 21, and periodically during the procedures. The offspring were weaned and separated into same-sex groups of four at PND21.

### Procedures

#### Control (CTRL) and prenatal alcohol exposure (PAE) without exercise

CTRL and PAE groups were housed for 30 days in a conventional cage (20.4 cm high, 45.4 cm long, and 23.4 cm wide).

#### Enriched environment (EE)

The enriched environment was based on Nithianantharajah and Hannan [58]. Animals of both groups (CTRL-EE and PAEE) were exposed to environmentally enriched cages (25 cm high, 79.4 cm long, and 50 cm wide), which have elements that promote physical activity: walls that allow climbing, wheels, tubes used as tunnels, chains such as climbing ropes, narrow rods for balance, unstable surface devices for walking. Food was left at an elevated point and a dark compartment was added to promote rest. As control, 2 groups of animals were housed in a cage of 20.4 cm high, 45.4 cm long, and 23.4 cm wide.

#### Endurance exercise (EEX)

The protocol was modified according to Bustamante et al. [55]. Animals PAE with endurance exercise (PAEX groups) were subjected to voluntary exercise sessions for 20 min for 4 weeks, 5 times a week. As an adaptation, the animals were exposed to the running wheel one week before starting the protocol. The wheel was not inside the cage to avoid alterations in the behavior of daily activities. The wheel had a revolution counter to measure the RPM reached for each animal.

#### Resistance exercise (REX)

This protocol was modified from Duncan et al. [26]. Animals of CTRL and PAE with resistance exercise (REX and PREX groups) climbed a 1-m ladder with an 85° incline designed to make the rats ascend into a dark chamber (20 × 20 × 20 cm) with a weight attached to their tails. It was performed in a series of 8 repetitions with a weight attached to their tails with 2 min of rest, 5 times a week on alternate days.

Weight for training was determined using a 1 RM (repetition maximum) test performed before training, which consisted of the animals climbing the ladder twice with 50% of their body weight attached to their tails. After completing the task, 30 g was added for another test (scale raised 2 times plus 2 min rest). This was repeated until the animals were unable to climb the ladder, and the weight was recorded as the maximum overload. The animals began the experiment by climbing with 50% of the maximum overload attached to their tails; each week, 10% of the maximum overload was added until they reached the maximum of 80% of the overload in the last week of the protocol.

### Measurement and information gathering techniques.

#### Muscle weight

After the protocol, the all groups were euthanized by isoflurane overdose and the medial (MG) and lateral (LG) gastrocnemius, extensor digitorum longus (EDL), soleus (SOL) and tibialis anterior (TA) muscles were carefully excised and weighed, were frozen, cooled in dry ice and then stored at − 80 °C for their conservation.

#### Agility

The measurement was obtained by Deacon [59]. Wooden bars of 8 mm were fixed every 60 cm in length so that the bar protruded horizontally. The end of the bar near the bench had a mark at 25 cm and another at 50 cm from the end to indicate the start and finish. The height of the rods from the ground was 60 cm. The time it took the rodent to make a 180° turn and the time it took to reach the other end was recorded.

#### Strength measurement

The protocol was used according to Deacon [23] as follows: strength at the beginning, middle, and end of the study was measured for each group. This physical capacity was evaluated by 2 tests of sustained contraction:

- Hanging time from the inverted mesh: the rodent was placed on a mesh to a 50 cm padded surface. The screen was reversed so the animal would hang. The time it took to hang on the mesh was recorded. There was a 60-s pause between each attempt and the average time that the animal managed to hold onto the mesh was calculated.
- Hanging time in horizontal bar. Animals were placed on a horizontal bar to 50 cm padded surface. The time spent on the bar was recorded. There was a 60-s pause between each attempt and the average time was calculated.

#### Coordination measurements (fingerprint test)

The protocol was used according to Carter et al. [60]. The front and hind legs were stained with non-toxic red vegetable ink. The animal was encouraged to walk in a straight line on paper placed in a corridor (80 × 15 cm). Fingerprint patterns were analysed, measuring stride length, the width of the front and rear base, and the overlap of the front and rear legs in centimetres.

#### Balance measurements

The protocol was used and modified according to Carter et al. [60]. The bar is 38 cm long and 49 cm across. The columns are fixed to a heavy wooden base. The bar diameters can vary by 2, 4, and 6 mm. Therefore, larger diameters have been included to refine the test as rats cannot generate proper grip. The time it takes the rat to get from one end of the bar to the other (80 cm of effective travel), the number of slides and stops it makes were recorded.

#### Irisin measurement

Plasma was obtained after a 5-min swimming period [61, 62]. Subsequently, the animals were anesthetized with isoflurane and sacrificed. Irisin was measured using a Phoenix Pharmaceuticals rat irisin (ELISA) kit [63]

### Data analysis

The data are presented as the means ± standard error of the mean (SEM). Data were analysed by the Kruskal–Wallis test was used to compare difference between groups in PND50, and the groups were compared with Dunn’s post hoc test. Mann–Whitney test was used for two unpaired samples and Wilcoxon test for two paired samples, to evaluate the differences between CTRL and PAE in PND21. Two-way ANOVA was used to evaluate differences between group in the test, and the groups were compared with Sidak post hoc test. Pearson correlation test was performed to determine the existence of any correlation between the revolution per minutes and the strength in group PAEEX. All statistical analyses were performed using GraphPad Prism V 5.0 software.

### Supplementary Information

Supplementary Material 1. Supplementary Figure 1. PAE affects agility and balance in PND21. Physical capabilities were altered in PAE condition versus CTRL. A. Time to go around the barr (CTRL and PAE group) B. Latency time to cross the barr of 8 mm (CTRL and PAE group) C. Number of stops during the balance test. D. Frontal stride length on footprint test. E. Hind stride length on footprint test. F. Front step width on footprint test. G. Hind step width on footprint test (N=4 animals per group). Data are mean ± SEM * p<0.05 ** p<0.01 (Mann-Whitney Test)

Supplementary Material 2. Supplementary Figure 2. Effects of endurance exercise on the strength of adolescent rats exposed to prenatal ethanol re-evaluated in PND50 and PND64. A. Endurance exercise scheme on wheel running B. Hanging Time in Mesh in PND21 and PND50. C. Hanging Time in Mesh in PND21 and PND64. D. Spearman’s correlation of turns per minute and time in the inverted mesh test in week 6 (each point represents a rat, where the average number of turns during the last week( R= 0,42.15, p= 0,28). N=4 animlas per group. Data are mean ± SEM * p<0.05 ** p<0.01 (Wilcoxon Test)

Supplementary Material 3. Supplementary Table 1. Muscle weight for animals in each group.

## Data Availability

The datasets used and/or analysed during the current study are available from the corresponding author on reasonable request.

## Abbreviations

CTRL: Control
CNS: Central nervous system
EDL: Extensor digitorum longus
EE: Enriched environment
EX: Exercise
FAS: Fetal alcohol syndrome
FASD: Fetal alcohol spectrum disorder
PAE: Prenatal alcohol exposure
PREX: Prenatal alcohol exposure with resistance exercise
PAEEX: Prenatal alcohol exposure with endurance exercise.
PND: Post-natal day
RPM: Revolution per minute
TA: Tibialis anterior
