# Supplementary figures and images for: Exercise reduces physical alterations in a rat model of fetal alcohol spectrum disorders
Source: Biol Res. 2024 Jun 22;57:41. doi: 10.1186/s40659-024-00520-2 (PMC11193177; doi:10.1186/s40659-024-00520-2)

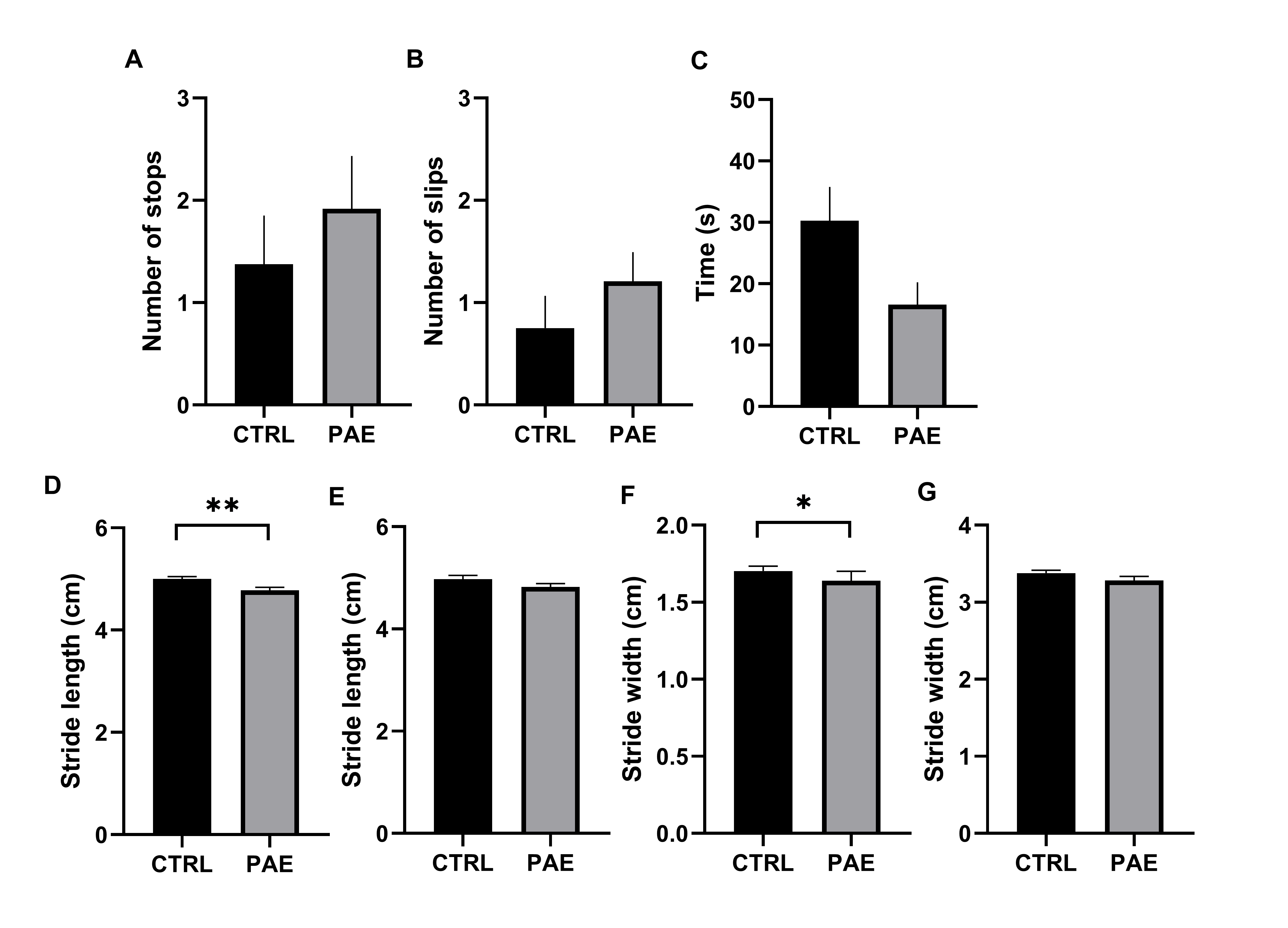

Supplement: Supplementary file 1 — Supplementary Material 1. Supplementary Figure 1. PAE affects agility and balance in PND21. Physical capabilities were altered in PAE condition versus CTRL. A. Time to go around the barr (CTRL and PAE group) B. Latency time to cross the barr of 8 mm (CTRL and PAE group) C. Number of stops during the balance test. D. Frontal stride length on footprint test. E. Hind stride length on footprint test. F. Front step width on footprint test. G. Hind step width on footprint test (N=4 animals per group). Data are mean ± SEM * p<0.05 ** p<0.01 (Mann-Whitney Test) [file 40659_2024_520_MOESM1_ESM.tif]

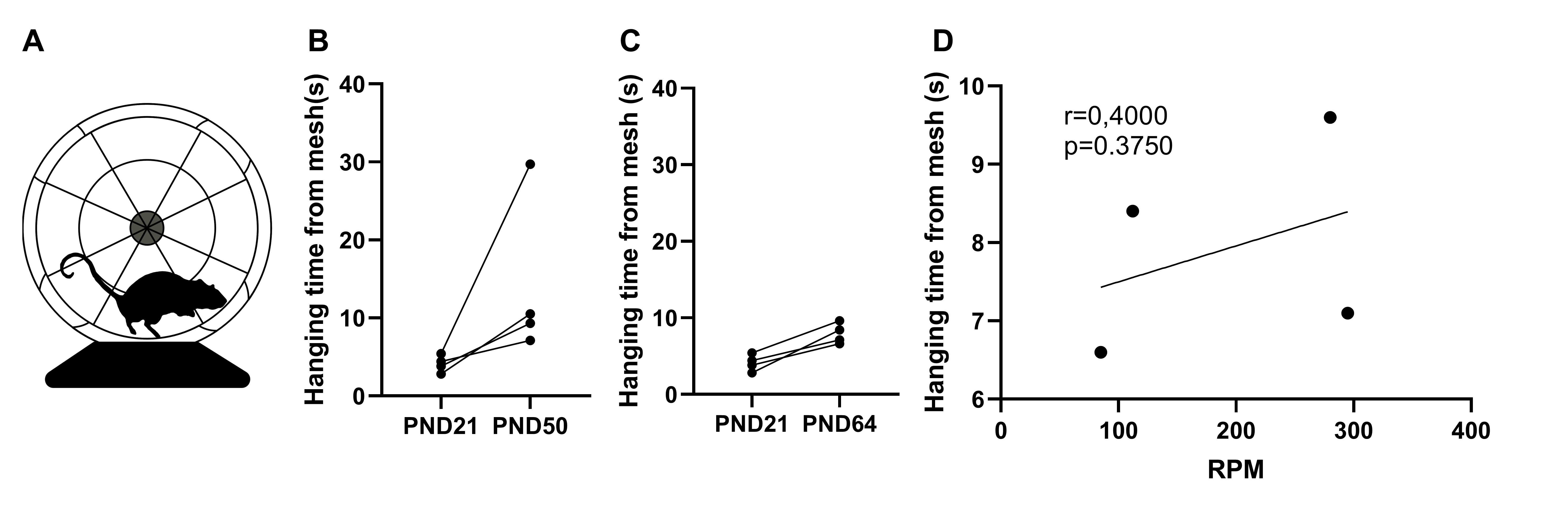

Supplement: Supplementary file 2 — Supplementary Material 2. Supplementary Figure 2. Effects of endurance exercise on the strength of adolescent rats exposed to prenatal ethanol re-evaluated in PND50 and PND64. A. Endurance exercise scheme on wheel running B. Hanging Time in Mesh in PND21 and PND50. C. Hanging Time in Mesh in PND21 and PND64. D. Spearman’s correlation of turns per minute and time in the inverted mesh test in week 6 (each point represents a rat, where the average number of turns during the last week( R= 0,42.15, p= 0,28). N=4 animlas per group. Data are mean ± SEM * p<0.05 ** p<0.01 (Wilcoxon Test) [file 40659_2024_520_MOESM2_ESM.tif]

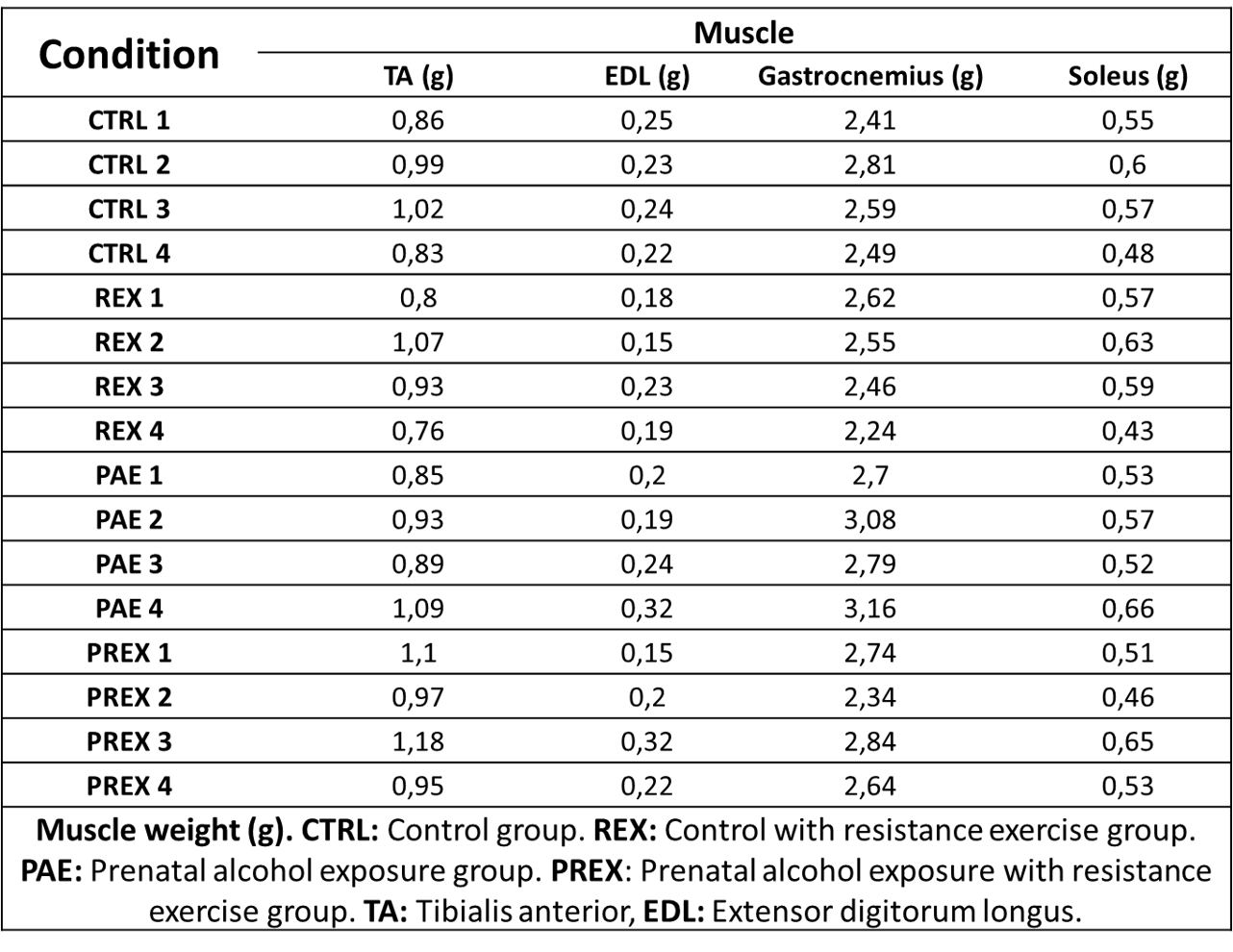

Supplement: Supplementary file 3 — Supplementary Material 3. Supplementary Table 1. Muscle weight for animals in each group. [file 40659_2024_520_MOESM3_ESM.tif]
